# ZIF-8 induced hydroxyapatite-like crystals enabled superior osteogenic ability of MEW printing PCL scaffolds

**DOI:** 10.1186/s12951-023-02007-w

**Published:** 2023-08-10

**Authors:** Bingqian Wang, Yuyang Zeng, Shaokai Liu, Muran Zhou, Huimin Fang, Zhenxing Wang, Jiaming Sun

**Affiliations:** Department of Plastic Surgery, Union Hospital, Tongji Medical College, Huazhong University of Science and Technology, Wuhan, 430022 China

**Keywords:** Zeolitic imidazolate framework-8, Biomimetic mineralization, Polycaprolactone, Melt electrowritten printing, Osteogenic differentiation, Bone regeneration

## Abstract

ZIF-8 may experience ion-responsive degradation in ionic solutions, which will change its initial architecture and restrict its direct biological use. Herein, we report an abnormal phenomenon in which ZIF-8 induces large hydroxyapatite-like crystals when soaked directly in simulated body fluid. These crystals grew rapidly continuously for two weeks, with the volume increasing by over 10 folds. According to Zn^2+^ release and novel XRD diffraction peak presence, ZIF-8 particles can probably show gradual collapse and became congregate through re-nucleation and competitive coordination. The phenomenon could be found on ZIF-8/PCL composite surface and printed ZIF-8/PCL scaffold surface. ZIF-8 enhanced PCL roughness through changing the surface topography, while obviously improving the *in-vivo* and *in-vitro* osteoinductivity and biocompatibility. The pro-biomineralization property can make ZIF-8 also applicable in polylactic acid-based biomaterials. In summary, this study demonstrates that ZIF-8 may play the role of a bioactive additive enabling the surface modification of synthetic polymers, indicating that it can be applied in in-situ bone regeneration.

**Supplementary Information:**

The online version contains supplementary material available at 10.1186/s12951-023-02007-w.

## Introduction

Zeolitic imidazolate framework-8 (ZIF-8) represents the subfamily of metal-organic framework (MOF) materials, which is comprised by organic ligands of imidazole derivatives and zinc metal ions (Zn^2+^). [1] Because of the chemical properties including stable structure, low cytotoxicity, and great specific surface area (SSA), ZIF-8 is used in different biomedical fields, [2–4] such as biological catalysis, [5, 6] biosensors, [7] and drug delivery system (DDS) [8, 9]. ZIF-8 has a tetrahedral architecture formed between imidazolate linkers and Zn atoms, and exhibits the hydrolytic stability. However, biological environment is extremely complicated, and some aqueous buffered systems have been usually adopted for *in-vitro* and *in-vivo* research. [10] ZIF-8 decomposition and collapse can be observed in phosphate buffered saline (PBS). Miriam et al. analyzed the mechanism associated with ZIF-8 degradation in PBS and discovered the affinity of phosphates for Lewis’s metal clusters. Therefore, it altered the coordination equilibrium for forming insoluble zinc phosphates, which could thus enhance 2-methylimidazole (2-HmIM) release. [11] In addition, competitive binding possibly promotes the exchange of ZIF-8 with anion in additional mediums containing abundant inorganic anions and metallic cations. [12].

Calcium phosphate (CAP), is the biomineral extensively adopted in our daily life, which offers the suitable environment for bone mesenchymal stem cells (BMSCs) in the process of bone repair. [13, 14] Thus, CAP coating can be adopted for improving the performance of bone tissue engineering scaffold. CAP coating with simulated body fluid (SBF) has been identified as the candidate method for providing appropriate temperature, ion content and pH for cells like the environment in human blood plasma. [15] In comparison with phosphate buffer saline, SBF solution displays great amounts of active metal cations and phosphates. Attached crystals and charged substrate regions within SBF are the nucleation sites that facilitate crystal growth through the conversion of high Ca^2+^, PO_4_^3−^, together with amorphous calcium phosphate contents in regular carbonated apatite, like apatite discovered within native bones. [16] Therefore, it is necessary to develop one strategy to promote the uniform apatite deposition generation within an adequate thickness onto polymer materials with no functional groups or charged regions, to promote mineralization of ions.

Among the improved bone grafting techniques, 3D-printed polymer bone scaffolds are considered as the temporary extracellular matrix alternatives for bone tissue engineering. [17] Polycaprolactone (PCL) represents the semi-crystalline and aliphatic polymer with suitable elasticity, comparatively slow biodegradation, low melting point (60 °C) and low inflammatory responses. [18] Due to the semi-crystalline and thermoplastic characteristics, PCL can well mix with additional materials; besides, it can be extruded to be filaments to achieve melt electrowritten (MEW) printing. Nevertheless, PCL is lowly bioactive, as a result, it has comprised cell attachment, osteoinduction and osteoconduction. [19] MEW printed scaffolds with gradient pore size and offset are reported with dense architectures, high porosity as well as mechanical integrity to facilitate cell infiltration for bone tissue engineering, [20–22] but it did not promote osteogenesis as expected. This calls for the development of strategies to modify MEW printed PCL scaffolds in order to increase bone regeneration and cell compatibility.

**Fig. 1:**
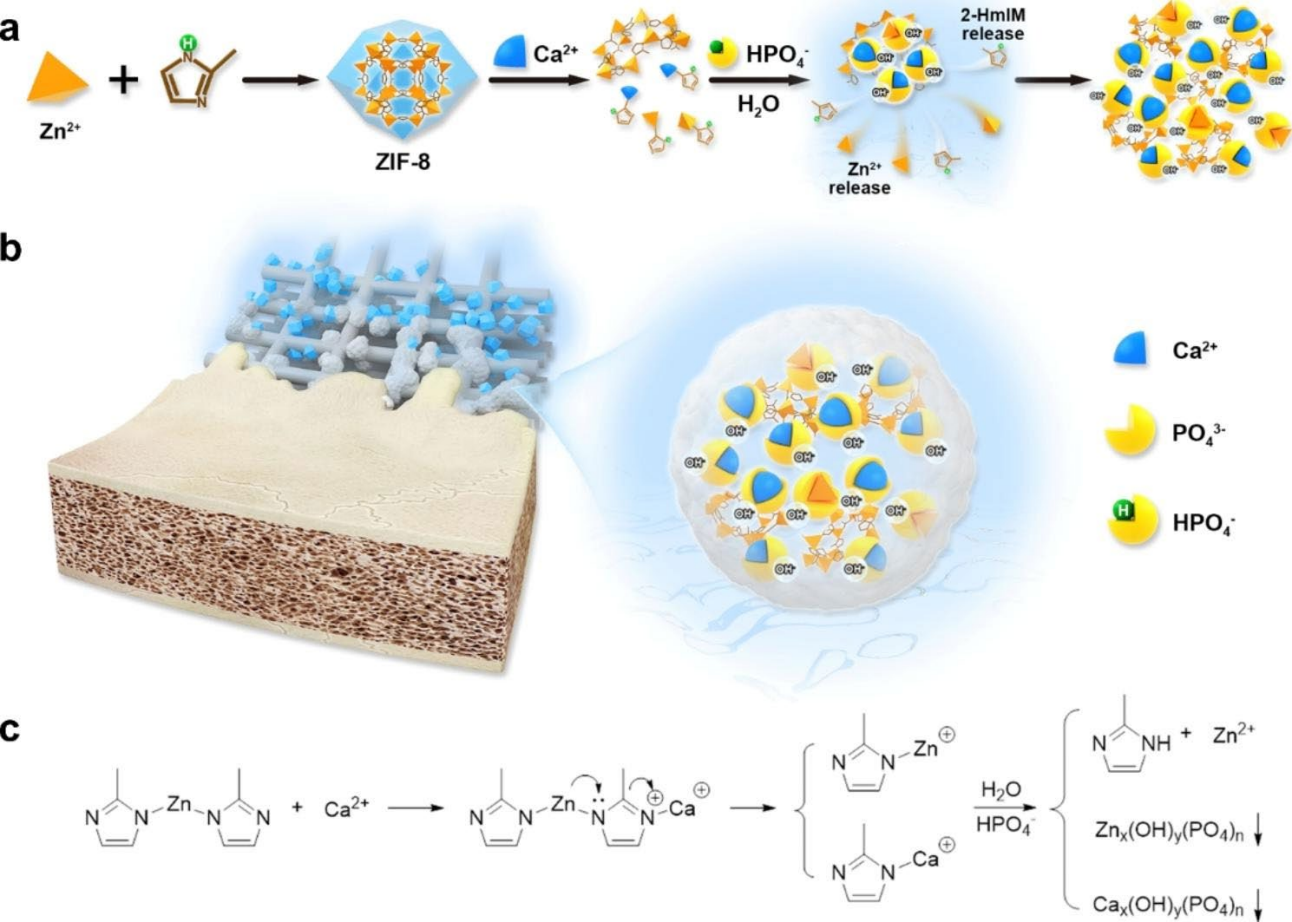
Schematic illustration of the mineralization process of ZIF-8 for enhancing bone regeneration. **a**) ZIF-8 mineralization under humoral environment. Ca attack induced ZIF-8 degradation, and this provided the Ca-P binding sites and caused apatite deposition. **b**) Mineralized ZIF-8/PCL scaffold prepared through melt electrowritten printing was implanted in the rat critical-sized skull defect to evaluate the bone regeneration enhancement effect. **c**) Hypothetical chemical reaction equations used to calculate ZIF-8 mineralization

In this study, association of ZIF-8 degradation with metallic cations/inorganic anion competitive binding within SBF was suggested to promote biomimetic PCL mineralization. Consequently, mineralized ZIF-8/PCL scaffolds were prepared using MEW printing technology for bone regeneration (Fig. 1). Thereafter, we analyzed MEW printed scaffolds in terms of their composition and morphologies. Besides, rBMSCs cultures were adopted to examine the osteoconductive performances and biocompatibility in vitro. Additionally, the present study constructed the rat skull critical-size bone defect model for evaluating mineralized ZIF-8/PCL scaffolds’ bone repair effects. We found that the ZIF-8 provided poorly biologically active materials with active sites and induced the osteogenesis-promoting potential in these materials in bone tissue engineering.

## Results

### Biomimetic mineralization and osteogenesis induction capacity triggered through ZIF-8 in vitro

**Fig. 2:**
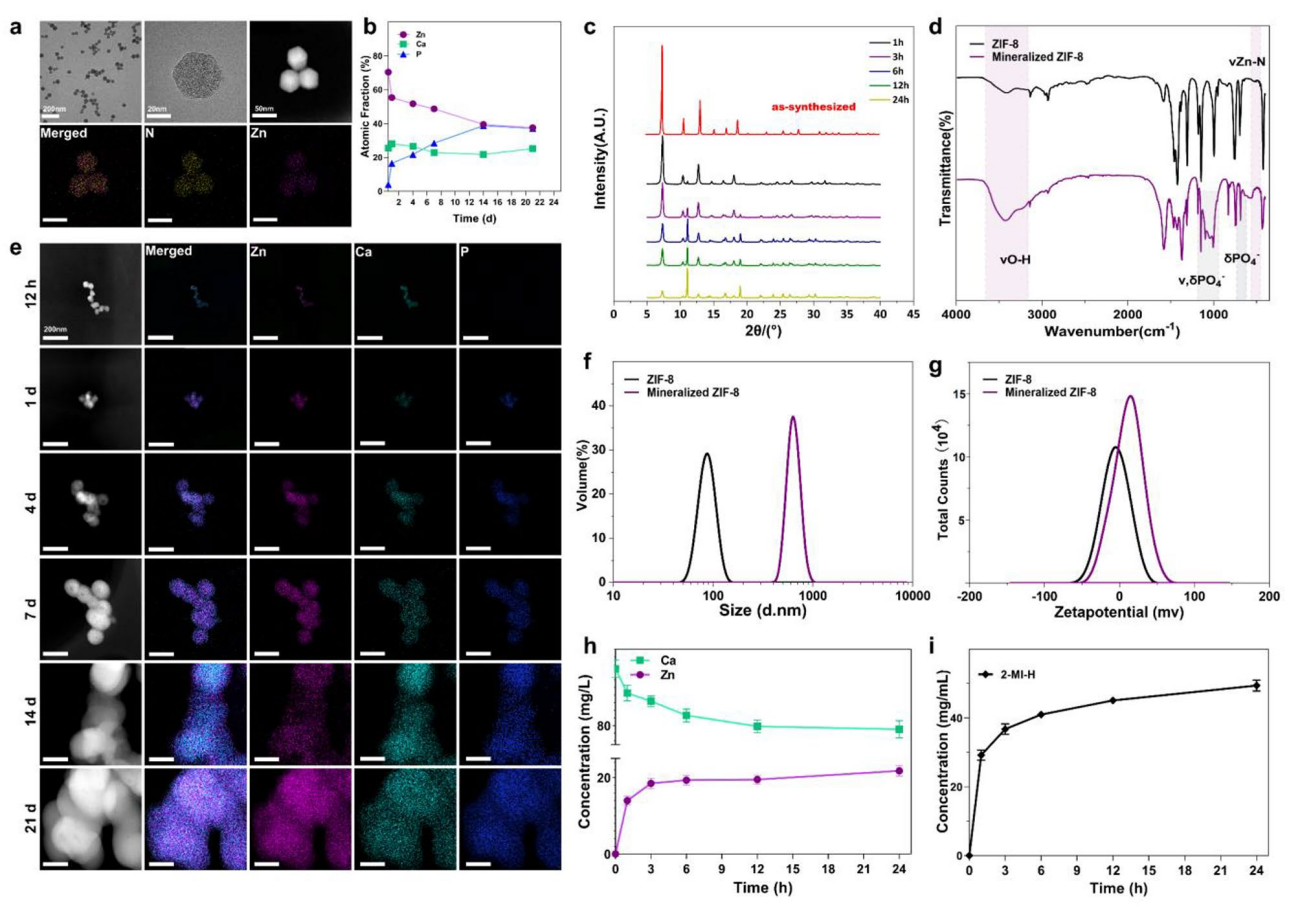
The process of ZIF-8 inducing biomimetic mineralization spontaneously. **a**) SEM and TEM-EDS analysis results presented that ZIF-8 particles had characteristic rhombic dodecahedral architecture and Zn-N skeleton elements. **b**). Quantification of elemental mapping for mineralized ZIF-8 NPs in panel e. **c**) XRD patterns displaying the pure phase for our prepared ZIF-8 (red line). ZIF-8 particles experienced partial collapse in SBF over time. Novel diffraction peaks appeared, suggesting that novel crystal structures were formed. **d**) By comparing FTIR spectroscopy on ZIF-8 particles prior to and following incubation in SBF (day 21), ZIF-8 degradation was related to ligand release and ion coordination environment alteration. **e**) TEM-EDS mapping images for ZIF-8 particles revealed the changes of composition and morphology in SBF as time goes on. Mineralized ZIF-8 possessed Ca, P and Zn elements as well as distinct agglomeration. **f**) ZIF-8 particle size following mineralization (day 21) obviously increased in relative to the initial size. **g**) Following mineralization (day 21), ZIF-8 had changed zeta potential and distribution from negative to positive. **h**) Zn^2+^ and Ca^2+^ contents in SBF suggested that Zn ions were released and bound to Ca ions in incubation. **i**) Quantification of 2-methylimidazole content in SBF revealed that ligand was released through protonation

A one-pot approach was utilized for synthesizing ZIF-8 nanoparticles (NPs). The prepared ZIF-8 NPs displayed characteristic the rhombic dodecahedral structure, and its size was even at 50 nm (Fig. 2a). ZIF-8 NPs were subjected to incubation within SBF solution for 12 h, together with 1/4/7/14/21 d under physiological conditions. Then, TEM was employed to analyze the extracted solids. The original rhombic dodecahedral structure was changed to spherical NPs clusters, followed by agglomeration and progressive enlargement. Based on subsequent EDS mapping, as volume elevated, incorporation of certain P and Ca atoms was observed in the homogeneous agglomerate (Fig. 2e). According to mapping images, atomic fractions for Zn, P and Ca exhibited the changing elemental distributions (Fig. 2b). Then, after SBF processing, XRD analysis was carried out to evaluate ZIF-8 particles in terms of relative crystallinity (Fig. 2c, Figure S1). Peak intensity on (011) plane of sod-ZIF-8 crystal showed a decreasing trend with increasing SBF immersion duration, indicating the defective ZIF-8 crystal architecture. Novel diffraction peaks were found, which demonstrated that novel crystal structures were formed. Similar results were observed in ICP-OES and GC-MS analyses (Fig. 2h, i). This work also carried out GC-MS for measuring MeIm contents, which displayed rapid protonation and release into the mother liquors. Ca deposition and Zn release were also analyzed by ICP-OES.

After SBF treatment, alterations of ZIF-8 atomic connectivity were examined by FTIR spectroscopy (Fig. 2d, Figure S1). Based on the spectra, peak intensities in vibration modes progressively declined associated with (ν_C=N_, 1584 cm^–1^; ν_ring_, 1500–1350 cm^–1^), while band intensity associated with Zn–N stretching mode (421 cm^–1^) progressively decreased. Those wide bands detected at 1160–900 cm^–1^ could be associated with PO_4_^3–^ antisymmetric stretching groups were highly affinal for polyvalent cations, it was speculated that the novel bands could be related to biomimetic apatite deposition as well as few zinc phosphates formation, which were ZIF-8’s degradation by-products. The above results revealed ZIF-8 NPs mineralization in the process of degradation.

DLS was adopted for measuring sizes together with zeta potentials (Fig. 2f, g). Following the biomimetic mineralization, mineralized mixture volume elevated. ZIF-8 had negative zeta potential, but mineralized ZIF-8 had positive zeta potential.

### In vitro osteogenesis potential assessment of mineralized ZIF-8

**Fig. 3:**
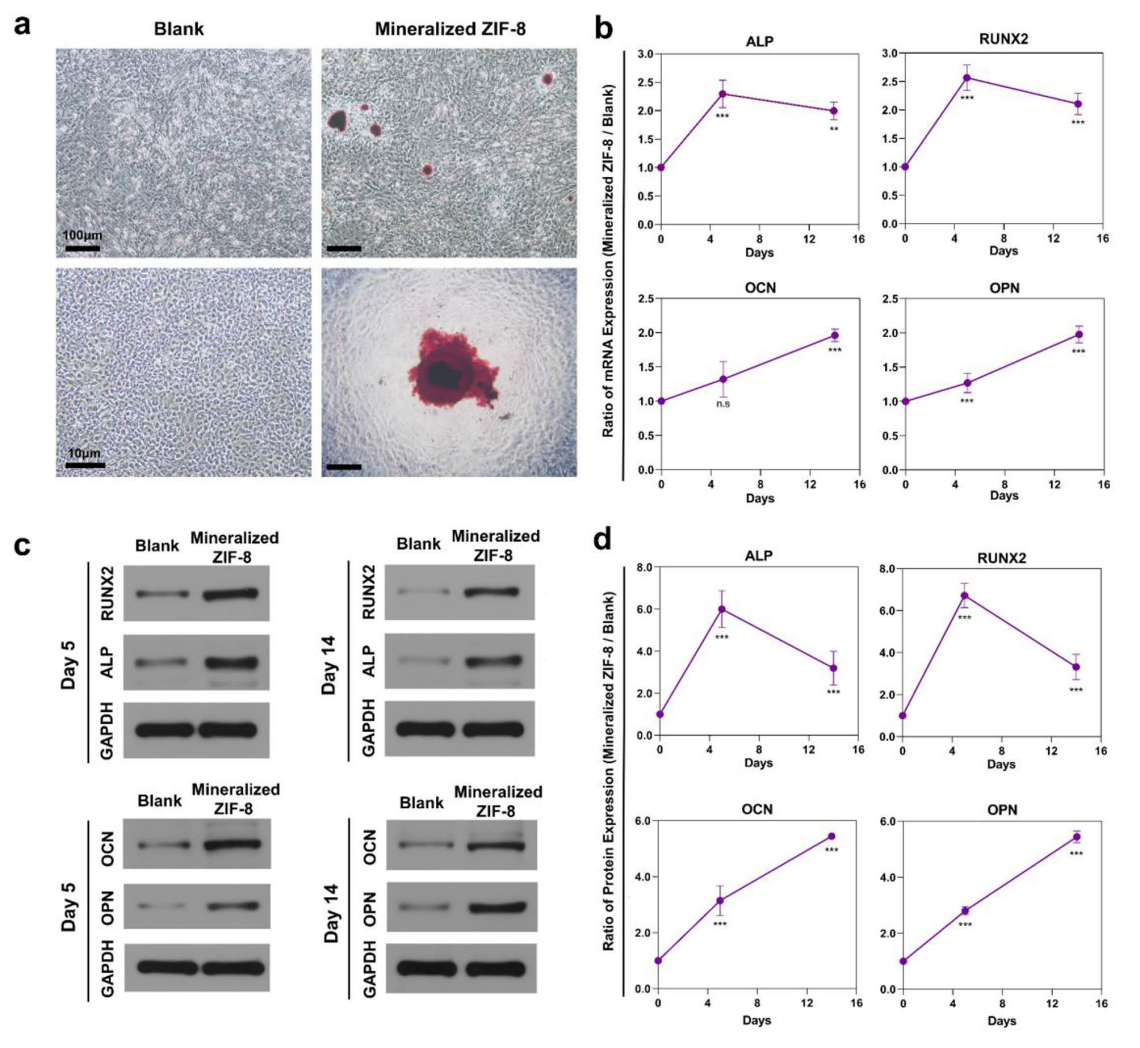
In-vitro osteogenic induction experiments based on rBMSCs co-cultured using mineralized ZIF-8 in the osteogenic induction solution-free normal medium. **a**) Typical digital images showing rBMSCs subjected to incubation using mineralized ZIF-8 showed the obvious mineralization ECM that contained mineralized crystalloid after 14 days, in comparison with rBMSCs subjected to incubation in normal medium (Alizarin red S staining.) **b**) Early (*Runx2*/*Alp*) and late (*Ocn*/*Opn*) markers for osteogenesis were remarkably up-regulated within rBMSCs after 5 and 14 days compared with those subjected to mineralized ZIF-8 incubation. **c, d**) WB images and summary analysis demonstrated the identical RUNX2/ALP/ OCN /OPN RUNX2 protein up-regulation trend. ** p < 0.01, *** p < 0.001, ns: not significant (n = 3)

According to optical microscopy following Alizarin Red S staining, rBMSCs incubated using mineralized ZIF-8 in the osteogenic induction solution-free medium showed mineral nodules after 14 days, which were not observed in control group (Fig. 3a). In addition, we also have previously verified that the mineralized ZIF-8 particles were unstained during the process. BMSCs co-cultured using mineralized ZIF-8 were collected to assess the reactions upon osteogenic differentiation-associated genes and proteins. Osteogenic marker levels were analyzed at 5-/14- days later. Relative to the controls, alkaline phosphatase (*Alp*), Runt-related transcription factor 2 (*Runx2*), osteocalcin (*Ocn*), and osteopontin (*Opn*) mRNA expression increased (Fig. 3b). Moreover, their protein levels elevated in mineralized ZIF-8 group (Fig. 3c, d). Therefore, mineralized ZIF-8 contributed to osteogenic differentiation induction in the osteogenic induction solution-free medium.

### Characterization of ZIF-8/PCL composite materials and printed ZIF-8/PCL scaffold

**Fig. 4:**
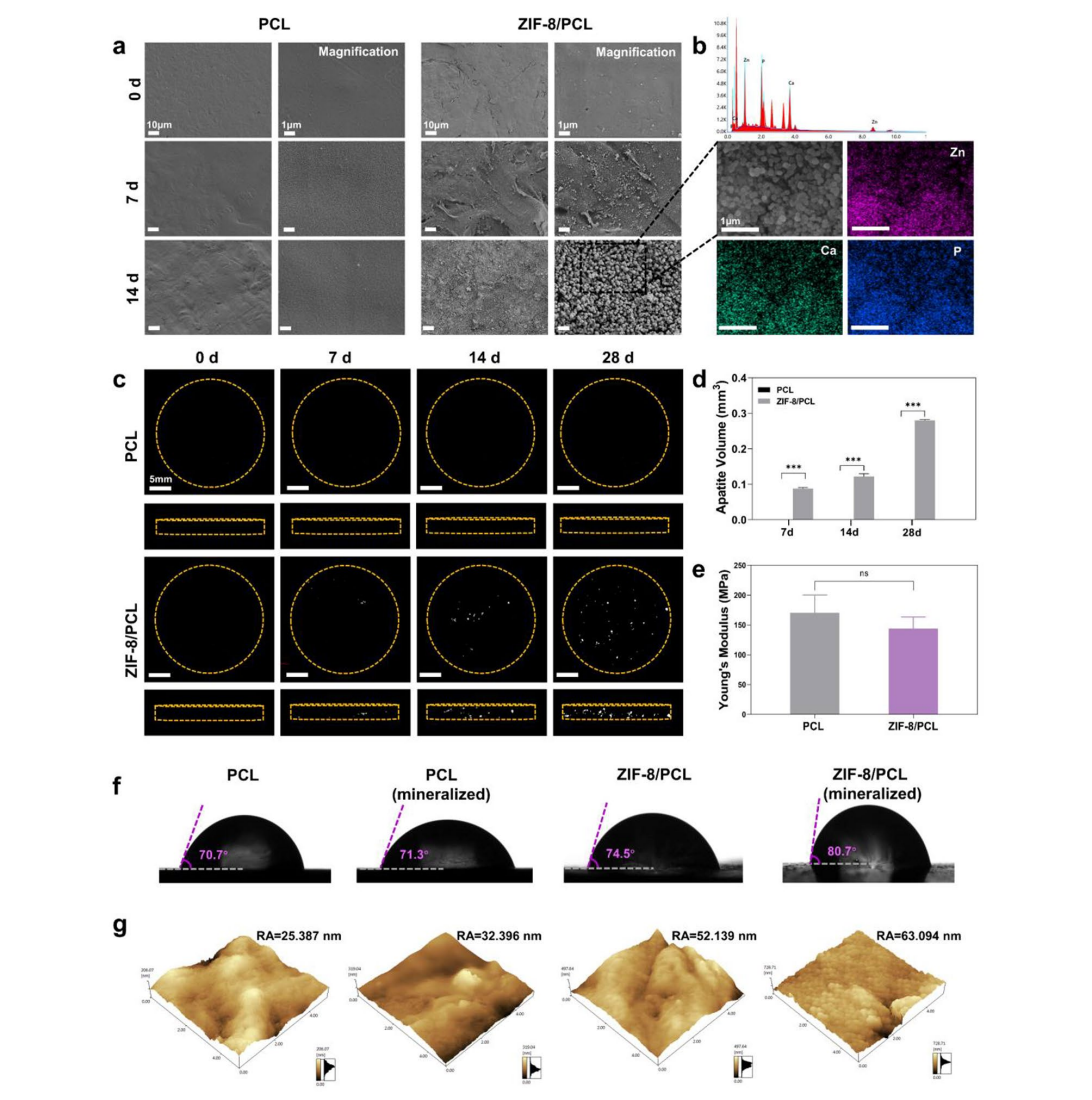
Characterization of ZIF-8/PCL composite after biomimetic mineralization. Flake-like PCL and ZIF-8/PCL prepared within the cylindrical mould. **a**) SEM images showed that crystals substantially deposited onto flake-like ZIF-8/PCL surface following SBF incubation under high and low magnifications. **b**) According to ZIF-8/PCL elemental mapping, crystal deposits included Ca, Zn and P elements. **c**) 3D micro-CT images for flake-like ZIF-8/PCL reconstruction revealed that bone-like apatite crystals deposited increasingly as time went by. **d**) Quantification on the ratio of apatite volume to total volume acquired based on stereograms. **e**) Young’s modulus of PCL was compared with ZIF-8/PCL, which revealed no obvious heterogeneity, and PCL was likely to become flexible following blending using ZIF-8. **f**) Static water contact angle was measured, as a result, adding ZIF-8 enhanced PCL surface roughness, especially following 7-day mineralization. **g**) AFM images revealed alterations of roughness following 24-h mineralization at the nanoscale. *** p < 0.001, ns: not significant. (n = 4)

After identifying the mineralization performances and associated mechanisms, we could fabricate flake-like PCL, ZIF-8/PCL through melt-mixing and compression molding synthesis. Following immersion in SBF for 7 and 14 days, SEM images suggested numerous crystal deposits onto ZIF-8/PCL surface (Fig. 4a). According to EDS-mapping, crystal deposits were constituted by Ca, P and Zn elements (Fig. 4b). Coronal-section and cross-section micro-CT images presented that apatites were attached onto surfaces over time (Fig. 4c). Quantification of apatite volume (AV) confirmed the deposition of apatites onto ZIF-8/PCL, whereas nearly no deposit was found in PCL (Fig. 4d).

We carried out compression test to evaluate PCL and ZIF-8/PCL concerning their mechanical performances (Fig. 4e, Figure S2). For PCL, its Young’s modulus was found to be 170.4 ± 15.92 MPa. As for ZIF-8/PCL, its value was measured to be 144.1 ± 9.67 MPa. The obtained findings implied that addition of ZIF-8 caused smaller elasticity modulus whereas elastic deformation in the presence of identical load increased. Water contact angle was also measured, verifying hydrophobic and hydrophilic degrees. There was no significant difference in water contact angle between mineralized PCL and PCL groups (71.3 ± 0.46° and 70.7 ± 0.65° separately). Because of ZIF-8 addition and the biomimetic activities, ZIF-8/PCL had the water contact angle of 74.5 ± 0.75°, whereas mineralized ZIF-8 had that of 80.7 ± 1.45°. (Fig. 4f). Obviously, AFM images on sample surface revealed surface roughness at the nanoscales. Based on the above findings, ZIF-8-mediated apatite deposition enhanced PCL surface roughness through a factor of two (Fig. 4g). This indicated that ZIF-8 combined with PCL significantly promoted PCL biomimetic mineralization, while the latter might not induce deposition of apatite independently.

**Fig. 5:**
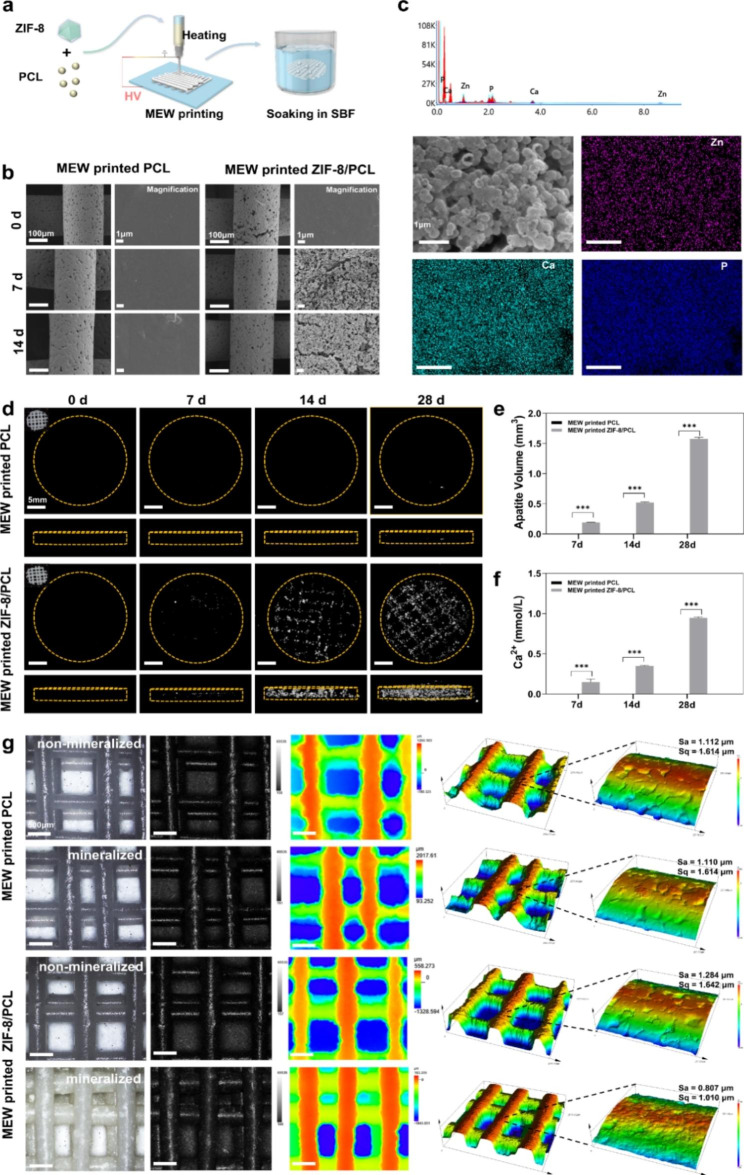
Characterization of MEW printed ZIF-8/PCL scaffold after biomimetic mineralization. **a**) Sketch map showing PCL and ZIF-8/PCL scaffold preparation through MEW printing. **b**) SEM images displayed thick crystal deposition lays onto MEW printed ZIF-8/PCL scaffold surface following SBF incubation, but no similar phenomenon was seen onto MEW printed PCL surface. **c**) Based on elemental mapping analysis, crystal deposition included Ca, Zn and P elements. **d**) 3D micro-CT reconstruction images revealed distinct bone-like apatite crystal depositions as time went by. **e**) Quantification on apatite volume/total volume ratio acquired based on stereograms. **f**) Ca quantification for diverse groups on the 28th day verified that Ca existed within the deposits onto MEW printed ZIF-8/PCL scaffold surface. **g**) LSCM images of ZIF-8/PCL and MEW printed PCL surfaces showed changes in surface roughness prior to and following mineralization at the micro-scale. *** p < 0.001 (n = 4)

Then, MEW printing was used to prepare PCL and ZIF-8/PCL scaffold (Fig. 5a). SEM was conducted to evaluate porous scaffold morphology (Fig. 5b). Microporosity was observed following printing, while ZIF-8 incorporation made no difference to the microporous structure of scaffold. Under the higher magnification (10000x), ZIF-8/PCL scaffolds with surface roughness exhibited high crystallinity after mineralization, which was different in relative to PCL with smooth surface. EDS mapping of elemental distributions of Ca, P and Zn demonstrated that apatites deposited homogeneously and uniformly in scaffolds (Fig. 5c). Moreover, Micro-CT analysis was performed to visualize and analyze 3D reconstruction images and uniform apatite layer distribution (Fig. 5d). As confirmed by quantification on apatite volume (AV) as well as Ca concentration, apatite depositions gradually grew on surfaces of scaffold fibers, (Fig. 5e, f). When compared with flake-like ZIF-8/PCL materials, more apatite was grown on the surface of MEW printed ZIF-8/PCL scaffolds.

The laser scanning confocal microscope (LSCM) was performed to analyze surface topography and 3D micrographs, showing surface morphological changes (Fig. 5g). In the presence of LED light, mineralized ZIF-8/PCL group showed significantly different color compared with the rest groups, which was even white and bright because of apatite deposits. In order to analyze roughness, the average roughness (Sa) and root mean square roughness (Sq) were measured. At the micron scales, PCL scaffolds had original Sa and Sq of 1.112 and 1.614 μm, respectively, whereas ZIF-8/PCL scaffold had the values of 1.284 and 1.642 μm, respectively. Following 7-day immersion in SBF, mineralized PCL scaffold roughness remained almost unchanged. Surface roughness of mineralized ZIF-8/PCL scaffolds decreased with the constant filling of surface micropores with the deposits (Sa = 0.807, Sq = 1.010).

### Biocompatibility testing of mZIF-8/PCL scaffolds in vitro

**Fig. 6:**
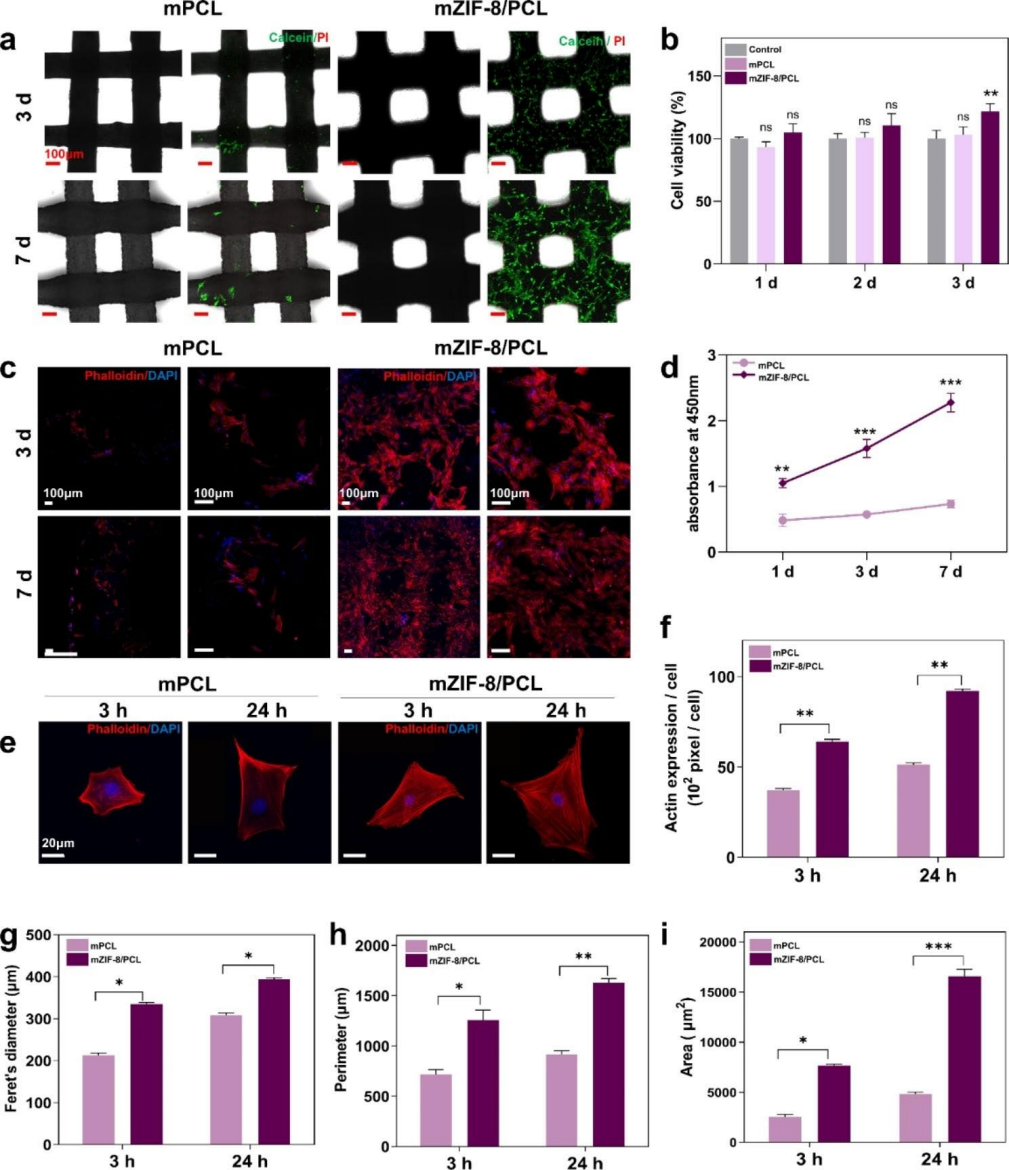
Biocompatibility for rBMSCs cultured on mineralized ZIF-8/PCL (mZIF-8/PCL). **a**) LSCM images showing rBMSCs inoculated on mPCL and mZIF-8/PCL for 3/7-day after Calcein-AM/PI staining. Obviously, mZIF-8/PCL exhibited the superior affinity for cells. **b**) Difference in cell cytotoxicity of rBMSCs co-cultured onto both groups was not significant in relative to control group. **c**) rBMSCs morphologies and quantities compared after culture for 3 and 7 days. Red and blue indicate actin filaments and cell nuclei, respectively. **d**) Cell viability analysis demonstrated the increased rBMSCs proliferation after co-culture using mZIF-8/PCL. **e**) rBMSCs inoculated on mZIF-8/PCL at 3/24-h post-culture displayed the superior cytoskeletal development, greater cell spreading area and formation of thick stress fibers. **f**) Cytomorphometry analysis based on images displayed in panel c. **g-i**) Action expression explored from the images. Actin expression in each cell and cell area was analyzed through densitometry based on confocal microscopic images shown in panel e. *p < 0.05, **p < 0.01, *** p < 0.001 (n = 6)

After MEW printed ZIF-8/PCL scaffolds were characterized, we explored cell viability and cell adhesion morphologies on diverse scaffolds. All scaffolds were immersed in SBF for a week in advance. In addition, we will use shorthand names, mPCL (mineralized PCL) and mZIF-8/PCL (mineralized ZIF-8/PCL), for the sake of brevity. Cell viability was analyzed using Live/Dead staining, DAPI and phalloidin staining. Before LSCM imaging, Calcein-AM and PI were added to stain rBMSCs inoculated on mPCL and mZIF-8/PCL scaffolds (Fig. 6a). Cells in mZIF-8/PCL scaffold displayed adherence, fast and even distribution, proliferation and growth. Living cells were mostly ordered down the orthogonally printed fibers. Cells in mPCL scaffold group showed little proliferation and adhesion on the scaffolds. CCK8 analysis on cell proliferation (Fig. 6d) also confirmed the superior cell viability of rBMSCs on mZIF-8/PCL scaffold. Moreover, CCK8 assay was conducted to analyze the possible toxic activities. As shown in Fig. 6b, the two scaffolds did not significantly affect the viability of rBMSCs.

LSCM, DAPI and phalloidin staining were used to assess the morphologies of rBMSCs adhering to the scaffolds. Following 3- and 7-day culture, we observed actin cytoskeleton in rBMSCs inoculated on the scaffold (Fig. 6c). Therefore, mZIF-8/PCL scaffold was more biocompatible than mPCL scaffold. In addition, rBMSCs morphology images were acquired following 3- and 24-h cell culture. After being incubated for 3 h, cells on the surface of mZIF-8/PCL scaffold increased relative to those on mPCL scaffold (Fig. 6e). Following 24 h incubation, morphology of rBMSCs on the surface of mZIF-8/PCL scaffold surface was extensively spread, with a large number of actin filaments extending to cytoplasm. Cytomorphometric parameters, such as cell area, Feret’s diameter and perimeter suggested that the differences were of significance between both scaffolds, proving the above qualitative findings (Fig. 6g, h, i). According to image-based densitometry, actin expression significantly increased (1.5- and 1.8- fold at 3 and 24 h, respectively) in cells seeded on mZIF-8/PCL scaffold (Fig. 6f).

### In vitro osteogenic properties of mZIF-8/PCL

**Fig. 7:**
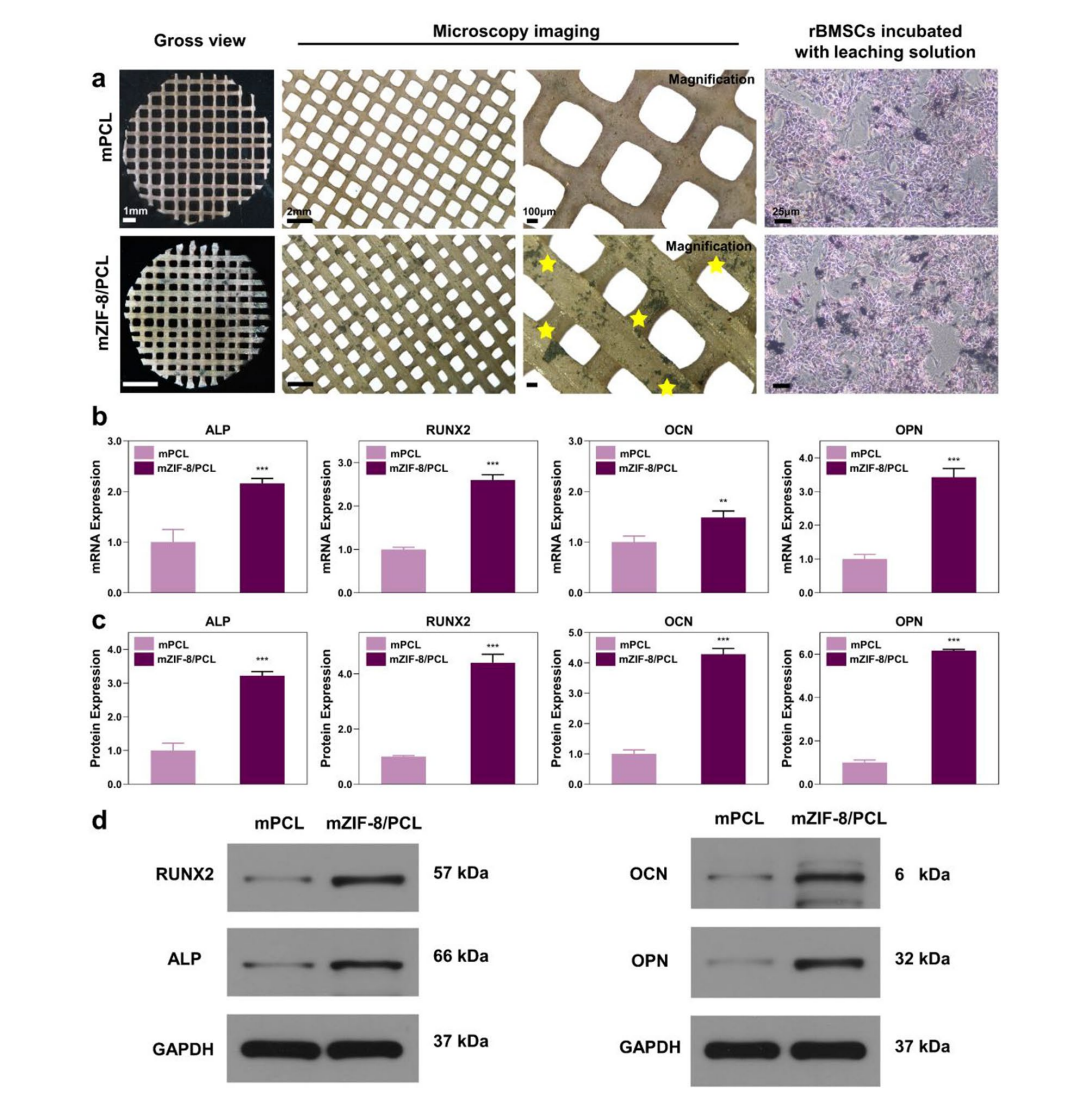
In vitro osteogenic properties of rBMSCs cultured with mZIF-8/PCL scaffolds. **a**) Macroscopic and microscopic observations on mPCL and mZIF-8/PCL after rBMSCs were cultivated within osteogenic induction medium and stained with Von kossa on the 14th day. Right-most panels: mineralization ECM of rBMSCs incubated using scaffold leaching solutions stained by von Kossa on the 14th day. mZIF-8/PCL group displayed a greater number of mineralized nodules from the two experiments. **b**) *Alp* and *Runx2* gene levels within rBMSCs on the 5th day following culture using mZIF-8/PCL together with *Ocn* and *Opn* gene levels within rBMSCs on the 14th day markedly elevated in comparison with mPCL group. **c, d**) Typical WB images and summary analysis revealed that RUNX2 and ALP expression elevated on the 5th day, while OCN and OPN expression elevated on the 14th day in ZIF-8/PCL group. **p < 0.01, ***p < 0.001 (n = 6)

As mZIF-8/PCL scaffold was found to exhibit cytocompatibility, this study further evaluated the ability of scaffold to promote osteogenesis. To facilitate the observation, osteogenic induction components were added into cell culture medium. ECM calcium deposits on the surface of scaffolds were stained with von Kossa to analyze formation of mineralized nodules. Calcified nodules were denoted in black. Cells inoculated on mZIF-8/PCL scaffold showed increased mineral deposition in relative to controls on day 14 (Fig. 7a). Using the scaffold leaching solution, similar results were obtained for rBMSCs cultured, indicating that zinc ions and other elements releasing from scaffolds also promote osteogenic differentiation. Through qRT-PCR, osteogenic gene expression was analyzed. mZIF-8/PCL scaffold group displayed significantly increased osteogenic gene levels relative to PCK and control groups, including *Alp*, *Runx2*, *Ocn*, and *Opn*, whose levels were elevated by 2.2-, 2.6-, 1.4, and 3.4-time, respectively (Fig. 7b). Furthermore, osteogenic protein levels of mZIF-8/PCL scaffold group increased in comparison with mPCL groups (Fig. 7c, d). As a result, mZIF-8/PCL scaffold promoted rBMSCs osteogenesis in vitro, mostly associated with ZIF-8 incorporation.

### Bone regeneration ability of mZIF-8/PCL scaffolds in rat cranial bone defect model

**Fig. 8:**
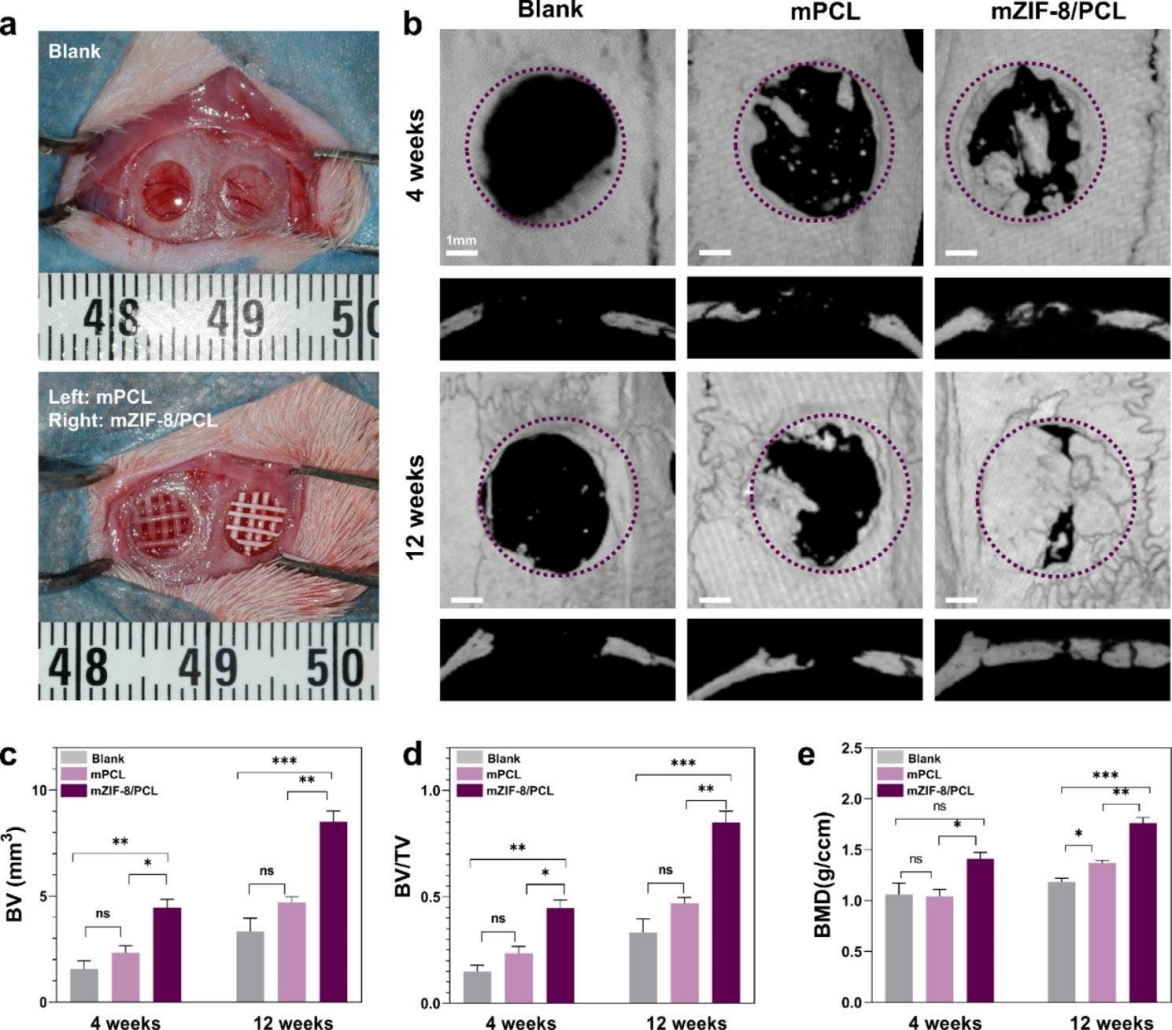
In vivo evaluation of new bone formation. **a**) Macroscopic observation of implantation area intraoperatively. **b**) Micro-CT 3D reconstruction images showing Ca bone defects at 4- and 12-week after implantation of mPCL and mZIF-8/PCL. Purple lines represent original cranial defect boundary. mZIF-8/PCL group had a greater number of new bones than the rest groups. Quantification of **c**) BV, **d**) BV/TV and **e**) BMD based on micro-CT analysis. * p < 0.05, ** p < 0.01, *** p < 0.001, ns: not significant (n = 8)

As mZIF-8/PCL scaffolds were suggested to show *in-vitro* osteogenesis, we further analyzed the *in-vivo* osteogenic effect using the rat calvarial defect models (5-mm in diameter) (Fig. 8a). Rat euthanasia was performed at 4- and 12-weeks postoperatively. Then, the defect locations were gathered, aiming to evaluate bone repair through micro-CT analysis and histology. Micro-CT images were reconstructed to reveal the new bones generated in bone defects (Fig. 8b-e). When analyzing osteogenesis, mZIF-8/PCL scaffold resulted in increased BV, BMD and BV/TV ratio. This enhanced new bone tissue growth and increased the average bone density in circular calvarial defect, in comparison with mPCL scaffold.

**Fig. 9:**
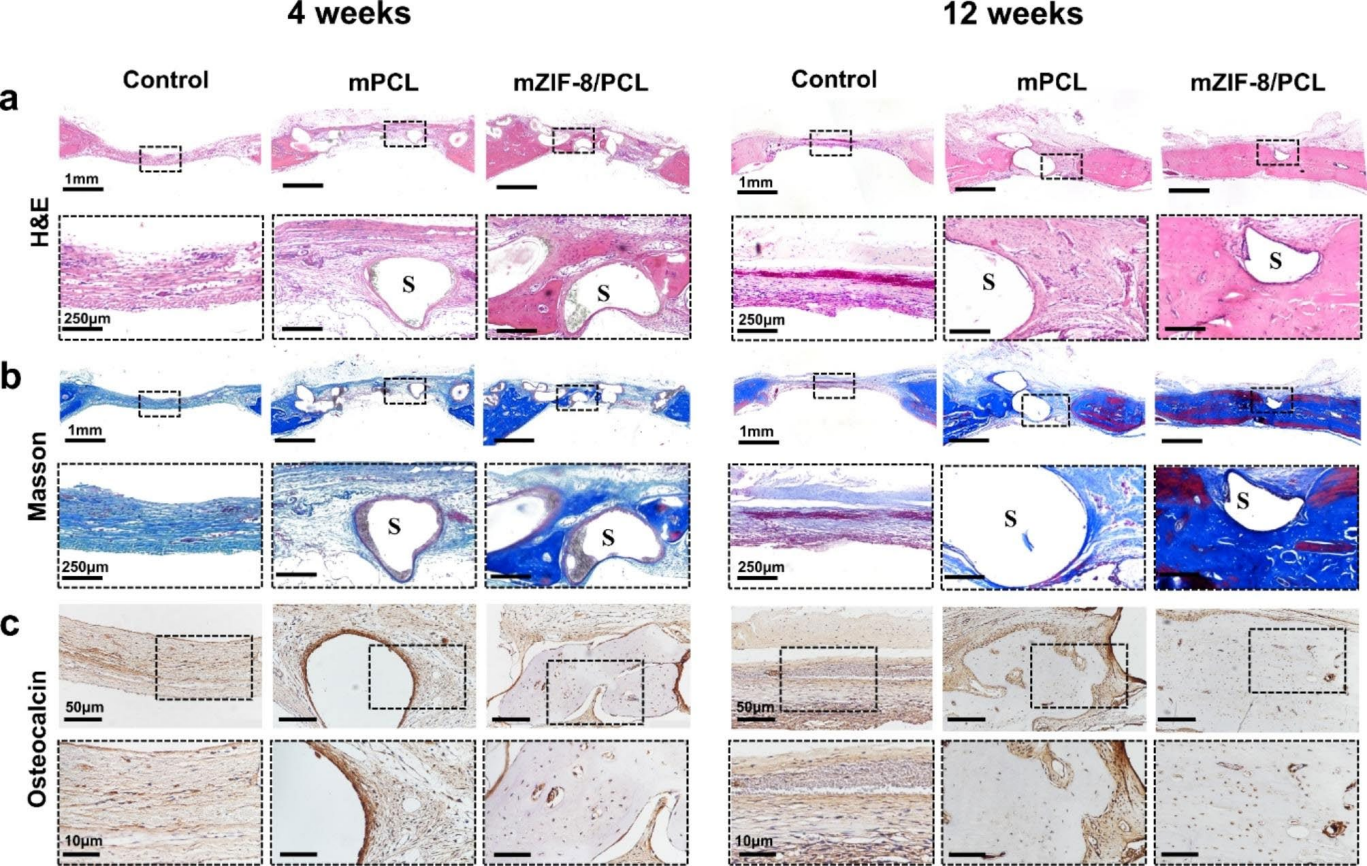
Histological and Immunohistochemical analysis. Representative osteogenesis histology analyzed following scaffolds implantation. **a**) Images of H&E staining, images of **b**) Masson’s trichrome staining. Black dotted rectangle indicates magnification images. S: scaffold. **c**) IHC analysis of OCN at 4-and 12-weeks following scaffold implantation

In this study, histological analysis was performed by H&E, IHC analysis and Masson’s Trichrome staining of decalcified bones, confirming the micro-CT observations (Fig. 9a, b). At 4-week post-implantation, new bone collected from defect edges and scaffold strut space of mZIF-8/PCL scaffold group suggested that scaffolds were highly osteoconductive. At 12-week post-implantation, defects in mZIF-8/PCL scaffold group were nearly completely closed. IHC analysis revealed the greatest OCN positively-expressed area of mZIF-8/PCL scaffold group relative to the rest groups (Fig. 9c). Histological analysis on H&E-stained major organs (cerebrum, kidneys and liver) was conducted to evaluate possible toxic activities, which revealed no alterations of organ pathology (Figure S3).

In addition, electrostatic spinning was performed to immerse ZIF-8 in SBF following the combination with polylactic acid (PLA); thus, ZIF-8/PLA induced mineral deposition (Figure S4). Likewise, additional MOFs were further tested; however, MIL-100/PCL and UIO-66/PCL did not have such mineralization activity (Figure S5).

## Discussion

We focused on investigating ZIF-8 degradation-mediated deposition of biomimetic apatite in humoral environment, while the poorly biologically active materials resulted in biomimetic mineralization on surface. Therefore, the ZIF-8-incorporated porous scaffolds were shown by MEW printing technique. In the process of ZIF-8 degradation, the release of Zn ions and the competitive binding of Ca ions to phosphate ions can induce apatite deposition. ZIF-8 had improved property after it was blended with PCL materials displaying favorable mechanical performances but low biological activity. In addition, ZIF-8/PCL materials have been suggested with favorable effect on promoting osteogenesis and inducing apatite deposition.

Due to the favorable thermochemical stability, pH sensitivity and insignificant cytotoxic effect, ZIF-8 can be the superb material used in bone regeneration. In addition to being used as the carrier in drug delivery in studies regarding biological activity during bone tissue engineering [8, 9], ZIF-8 is found to enhance osteogenesis by releasing Zn ions via degradation under humoral milieu [23–25]. Zn ion release is analyzed in terms of its impact, while products generated based on reactions of ZIF-8 degradation with additional solution ions are analyzed. Phosphate species can change ZIF-8 degradation mechanism in PBS that includes coordination equilibrium of Zn^2+^ ions with solution 2-HmIM. These phosphates are highly affinal for phosphate groups. Therefore, polyvalent cations can shift the equilibrium to the formation of inorganic by-products with low insolubility [11]. Different from PBS, iron environmental balance can be extremely complicated in SBF. SBF solutions include Ca ions, and they may generate Ca and P deposition through competitive combination in solutions. In this study, apatite deposition was found after ZIF-8 degradation and induction in SBF, which could eventually generate mineralized pellets. Zn-N bonds and hydrogen bonds in ZIF-8 slightly decompose because of Ca ion attack in SBF solution. Therefore, the intermediate containing Zn and Ca ions bonded with one individual 2-HmIM ligand was formed. Then, HPO_4_^−^ and H_2_O were hydrogen donors to induce rapid 2-HmIM protonation, resulting in the formation of excess calcium and zinc hydroxy phosphates. Such protonation process may induce partial Zn ion release. In addition, during the replacement process in SBF, the Ca contents were stably high, with precipitation equilibrium always shifting towards Ca hydroxy phosphate direction. Due to electrostatic adsorption and covalent bonding, continuous agglomeration of mineralized pellets was found. The variations in particle size found in TEM images are consistent with the DLS results, indicating great hydroxyapatite-like crystals formation and continuously increasing surface potential. During this process, Zn ions were released, Ca ions were used in reaction system, and excessive protonated 2-HmIM was also released. As a result, soluble hydrogen phosphate in SBF changed to precipitation-prone phosphate ions, causing the deposition of apatite. Hydroxyl groups could be identified in mineralized and non-mineralized ZIF-8 through FTIR, thereby promoting crystal development, electrostatic interaction-induced aggregation, and morphology [26]. Based on EDS mapping images, Zn ions showed uniform distribution in mineralized ZIF-8, probably due to that prior to bond breaking, partial ZIF-8 residues were covered with mineralized pellets in the process of degradation. Moreover, a low amount of insoluble zinc phosphate was generated in the process of ion competitive binding, even though Ca ions exhibited the increased reactivity in relative to Zn ions. Since apatite and zinc enhanced bone regeneration by promoting osteogenesis [8, 27–29], positive results of osteogenic experiments have been illustrated.

As for surface compositions of scaffolds, their surface components will impact the integration and response [30]. PCL, the commonly used biomedical polymer material, exhibits favorable biocompatibility, toughness whereas low cytotoxic effect when being used as the supporting material for scaffolds [31]. Nonetheless, PCL-only scaffolds do not possess any active surface coating or sufficient recognition sites to achieve bone binding, as a result, bone repair time will be prolonged [32].

To improve biomimetic mineralization characteristics and to retain mechanical characteristics, ZIF-8 was speculated to be appropriate for PCL. Therefore, flake-like ZIF-8/PCL was synthesized through melt mixing molding. The sample was immersed in SBF for a few days. As a result, obvious mineralized crystallization deposits could be found on sample surface. Bone regeneration is closely related to the equilibrium of scaffold architecture with mechanical performances. Scaffolds need to guarantee adequate porosity, offer the environment to penetrate vasogenic cells, facilitate adjacent tissue development, and prepare for mechanical strength generation like that in normal bone, aiming to ensure the efficacy of the implant [33]. MEW technology well regulates pore size and porosity of scaffolds [21]. The best stent aperture to regenerate hard tissues was previously reported to be 200–500 μm [34]. Therefore, by adjusting MEW machine parameters, we synthesized a scaffold around 150 μm in diameter with an inter-fiber aperture of 200–300 μm, and it retained mechanical strength and promoted tissue blood vascular development. Relative to flake-like ZIF-8/PCL, MEW printed ZIF-8/PCL scaffold displayed a larger number of apatite depositions in the identical immersion time. It might be due to the fact that MEW printing technique offered the three-dimensional (3D) scaffold with an increased surface area-to-volume ratio and facilitated apatite depositions. The melt formed the microporosity architecture in extrusion, thereby increasing scaffold surface area and sites of biological mineralization.

Anchoring dependent cell division is not achieved with no previous extension on the surface of growth substrate, and these cells may experience apoptosis [35]. Therefore, during early cell expansion, cell expansion area shows positive relation to cell proliferation. Based on our results, mZIF-8/PCL scaffold was found to trigger suitable cell responses regarding cell growth, attachment, and adhesion. To be specific, mPCL scaffold group had inferior cell spreading, actin organization and morphology. mZIF-8/PCL scaffold had superb cell activities, which was possibly associated with different aspects. The ZIF-8 particle surface possesses negative charges, while fibers also possessed negative charges in MEW printing process. Therefore, ZIF-8/PCL surface usually has negative charges, and it is beneficial for apatite nucleation [36]. In the process of mineralization, apatite layers with positive charges were slightly generated. zeta potential of ZIF-8 particles altered from negative to positive. As a result, mZIF-8/PCL scaffold was positively charged. Compared with negatively-charged surface, the positively-charged one achieved superior cell adhesion, since ECM molecules that mediate cell adhesion have negative charges, and they are more likely to attract positively-charged surface [37–39]. MEW printed scaffolds had gradient and offset pore size architecture, which were good for great porosity and mechanical integrity in order to facilitate cell infiltration during bone tissue engineering [21]. In addition, surface roughness is also another aspect. During biomimetic mineralization of microroughness, the roughness of mZIF-8/PCL surface increased, thereby enhancing deposition of apatite, and promoting original cell attachment, growth and differentiation [40, 41].

In addition, mZIF-8/PCL scaffolds significantly affected rBMSCs osteogenic differentiation in the co-culture system. Based on osteogenic induction experiments in the presence of mineralized ZIF-8, Ca nodule was found in a small amount, since ZIF-8 mineralization occurred prior to co-culture, and Zn release showed no relation to osteogenic induction, while Zn in biomaterials increased osteogenesis-related gene levels, including *Alp*, *Runx2*, *Col I*, *Opn*, and *Ocn*. Therefore, it promoted ECM mineralization by enhancing Ca deposition and Col secretion [25]. Although the underlying mechanisms of is still unclear, we might speculate that the most significant benefits were the following three events based on the above results: (1) The mineralized crystallization deposits layer induced by ZIF-8 spontaneously. The ability of in vitro apatite mineralization of biomaterials in SBF is usually considered as in vivo osteogenic activity. ZIF-8 can spontaneously induce strong biomimetic mineralization in line with the above reaction principle. (2) The formation of a roughened surface structure. Previous reports have indicated that surface roughness of the material is a contributing factor in enhancing the initial adhesion of BMSCs ^[[42, 43]]^. Furthermore, a direct and strong linear relationship has been observed between the expression levels of marker gene of bone and the surface roughness of the material [44]. (3) The release of Zn ions. The role of Zinc in bone metabolism has been established through cellular and molecular evidence. Incorporating Zn into biomaterials can enhance the proliferation of osteoblasts and the activity of ALP, affect the synthesis of osteopontin and osteocalcin, as well as activate the extracellular signal-regulated kinase pathway (ERK), causing changes in the expression level of *Runx2* and other osteogenesis-related genes ^[[45, 46]]^. As far as mZIF-8/PCL scaffolds were concerned, the interior ZIF-8 in PCL was exposed when PCL was degraded, which synergistically promoted osteogenic differentiation through Zn and biomimetic mineralization. However, this process required more verification. In vivo, mZIF-8/PCL scaffolds significantly enhanced the regeneration of bone defect, as evidenced by the new bone ingrowth near defect edge as well as between scaffold fibers, with significantly increased volume compared with control group. Nonetheless, mZIF-8/PCL scaffolds showed relationship to restricted bone regeneration. Firstly, MEW fiber diameter ranged from several to dozens of micrometers, which was obviously increased compared with nanoscale ECM. Secondly, slow PCL degradation probably avoids later osteogenesis. More research needs to be conducted to better analyze the possibility.

## Methods

### Synthesis of ZIF-8 particles

The ZIF-8 was synthesized as formerly reported with minor modifications. [47] Briefly, 1.68 g zinc nitrate hexahydrate within methanol (20 ml) was blended with 4 g 2-methylimidazole contained within methanol (40 ml), and the mixed solution was put onto the magnetic agitator for 2-h stirring at 650 r·min ^− 1^ under room temperature (RT). The Zn^2+^: 2-HmIM: methanol molar ratio within the reaction system was 1:10:30. Afterwards, the sample was centrifuged at 8500 rpm to separate ZIF-8 powders, followed by methanol washing as well as 24-h drying under 60 °C.

### ZIF-8-induced calcium and phosphorus deposition

ZIF-8 particles were immersed into SBF to evaluate *in-vitro* biomimetic mineralization under 37 °C within the thermostated container for specific time points, with replenishing of SBF solution at 2-day intervals. This was followed by washing with deionized water twice, as well as solid drying with the lyophilizer. NaCl, NaHCO_3_, KCl, K_2_HPO_4_•3H_2_O, MgCl_2_•6H_2_O, and CaCl_2_, Na_2_SO_4_ were dissolved in ddH_2_O for preparing SBF solution, with 1 M HCl solution and Tris (hydroxymethyl) aminomethane being used to adjust pH to 7.40 under 36.5 ℃.

### Characterization of the mineralized ZIF-8

#### Transmission electron microscopy (TEM) an element mapping

Analysis was carried out using Talos F200X (FEI) at the 200 kV accelerated voltage. Following 5 min of 2 mg/ml ethanol dispersion and sonication, TEM grid was adopted for deposition of obtained powders.

#### XRD and FT-IR analysis

X-ray diffract meter (XRD) was utilized to characterize obtained powders for their crystalline phases within the 5–40° 2θ range and at the 4.7°/min scanning rate. Fourier Transform Infrared (FT-IR) test was performed using FT-IR spectrophotometer (Thermo Nicolet).

#### Zn^2+^, Ca^2+^, and 2-methylimidazole content measurements

Following immersion of ZIF-8 (15 mg) within SBF (15 ml) for 1/3/6/12/24 h, this work collected supernatants to be the mother liquors. Thereafter, Zn content within mother liquors was analyzed through the axial viewing of ICP-OES (5110, Agilent). By using emission line of Zn (II) at 213.856 nm, GC-MS (7890 A/5975 C, Agilent) was adopted for analyzing mother liquors. In brief, an equivalent amount of (1 µl) solution was injected in the split mode under the 250 °C injection port temperature. Sample was later separated onto the pole from 60 °C to 1 min to 240 °C at the 15 °C min^− 1^ heating rate. Helium was utilized to be a carrier, and the downline rate was maintained at 35 cm/s under the constant current. In addition, quadrupole operation was conducted under the scanning mode within the 50–150 amu scanning range and 5 times/s scanning rate, with the interface and ion source temperatures being 230 and 200 °C, separately. Correction curve was constructed by the two-point external standard approach. Analysis was conducted three times.

#### Particle size and zeta-potential assessment

The dynamic light scattering (DLS) plus ZETA system (Malvern Zetasizer Nano S) was employed for analyzing ZIF-8 together with mineralized ZIF-8 with regard to particle size as well as zeta-potential.

### In vitro osteogenic differentiation assessment of mineralized ZIF-8

#### rBMSCs separation as well as in-vitro cultures

This work separated and collected rat bone marrow stromal cells (rBMSCs) according to previous description. [48] In brief, the 3-5-day-old neonate Sprague Dawley (SD) rats were given cervical dislocation for euthanasia, followed by 30-min immersion within 75% alcohol. Later, muscles as well as soft tissues attached onto femurs and tibias were removed, cartilages at the two bone terminals were cut, and cavities were rinsed by culture medium repeatedly till they were white. Later, freshly collected bone marrow was inoculated into 10-cm culture dishes that contained culture medium. Afterwards, dish incubation was carried out under 37 ℃ with 5% CO_2_ (Heracell150i, Thermo Scientific), with medium change at 3-day intervals. After r-rBMSCs achieved confluency, cell passage was performed.

#### Alizarin Red S Staining

We inoculated rBMSCs (1.5 × 10^5^) into 6-well plates for a 24-h period or grew then till reaching 60% confluency. Cell co-culture with 200ng/ml mineralized ZIF-8 was completed within the osteogenic induction solution-free culture medium for a 14-day period, followed by 30-min fixation within 4% paraformaldehyde (PFA). After removing fixation solution, cells were stained with Alizarin Red reagent for indicating ECM mineralization. Excessive Alizarin reagent was later discarded with DI water. Images were taken with the Nikon Eclipse Ts2R-FL microscope.

#### Osteogenic gene levels

In Real-Time PCR (RT-PCR), rBMSCs were co-cultured with mineralized ZIF-8 for 5/14 days, exposed to Trizol-up treatment to extract mRNA, and loaded for RT-PCR analysis with SYBR Green qRT-PCR kit (ELK Biotechnology) to analyze *Alp*, *Runx2*, *Ocn*, and *Opn* mRNA levels, with β-actin being the endogenous control.

#### Osteogenic protein expression analysis

To conduct cell protein immunodetection, Western-blot (WB) assay was conducted for determining osteogenic protein expression within rBMSCs. Total Protein Extraction Reagent (Aspen) was utilized for cellular protein separation on ice. Proteins were boiled within the loading buffer for an 8-min period under 99 °C, then protein aliquots were exposed to 12% SDS-PAGE for separation, followed by transfer on PVDF membranes (Millipore). Primary antibodies against RUNX2 (Abcam) and ALP (LSBio) were added to incubate membranes on day 5, whereas those against OCN (sc-390,877, 1:500, Santa Cruz) and OPN (ab63856, 1:1000, Abcam) were added to incubate membranes on day 14. Later, secondary antibodies (1:10000) were added to further probe membranes, followed by detection with chemiluminescence (ECL) (AS1059, Aspen). In addition, the ChemiDoc Touch chemiluminescent system (Biorad) was applied in protein visualization. GAPDH (ab181602, Abcam) served as the reference for normalizing osteogenic marker levels. According to gray value, image J was utilized to analyze target protein expression.

### Scaffold printing

The PCL and ZIF-8 mixture (10% wt.) after melting was mixed onto the 120 ℃-heating plate for a 30-min period. Following pressing, PCL and ZIF-8/PCL compacts could be acquired. MEW printing machine (QINGZI NANO) was later adopted for preparing melt electrowritten scaffolds. Later, PCL particles (3.6 g) were sufficiently blended with ZIF-8 powders (400 mg), then the mixture was put into the feeding tank for 30-min heating under 120 ℃. Printed MEW parameters included interval = 1 mm, atmospheric pressure = 0.18 MPa, speed = 8 mm/s, square wave = 20 times, as well as acceleration = 48 mm/s^2^. Besides, the charge polymer fiber was prepared by applying threshold voltage from − 5 to 7 kV. Samples were later sectioned to round sheets with the diameter and height of 5 and 2 mm separately.

### Evaluation of biomimetic apatite deposition on scaffolds

For accelerating biomimetic apatite deposition, CaCl_2_ and K_2_HPO_4_ solutions were added to immerse samples, followed by 3-min immersion of scaffolds into 0.2 M CaCl_2_ solution (20 mL), 10-s immersion within ddH_2_O (30 mL), then 3-min immersion within 0.2 M K_2_HPO_4_ solution (20 mL) and 10-s immersion within ddH_2_O (30 mL). This whole pretreatment process was conducted in triplicate. Those immersed samples were later soaked into SBF to deposit biomimetic apatite (in brief, 30 mL SBF was added in the 50-ml centrifuge tube that contained six immersed samples), followed by standing under 37 ℃ for 1/7/14 days, with SBF change every day for maintaining the identical ionic strength during this process. Each sample was eliminated out of SBF, slightly rinsed by ddH_2_O, and later lyophilized for a 24-h period under − 50 ℃ at vacuum.

### Characterization of scaffolds

#### Scanning electron microscopy (SEM) and EDS-mapping

Gemini 300 (Zeiss) was employed to record images. 15-s gold spaying was completed onto mold-made PCL, ZIF-8/PCL, as well as MEW printed PCL and ZIF-8/PCL samples, followed by scanning at 5 kV.

#### Micro-CT analysis

For exploring deposition of biomimetic apatite onto samples, this work utilized the Micro-CT scanner (SkyScan 1176; Broker), and the slice thickness ≈ 18 μm. Additionally, VG studio software (Volume Graphics GmbH) was employed for reconstructing 3D images. The micro-CT assistant software CTAn (Scanco Medical, Zurich) was applied in determining apatite volume (AV) and the AV-to-total volume ratio (AV/TV).

#### Calciumions content measurements

Briefly, mineralized samples were dissolved into 0.5 M acetic acid (0.4 mL) overnight and quantified by the calcium assay kit (BioAssay Systems) in line with specific protocols.

#### Mechanical performances

The Electronic Universal Testing Machine (AG-IC 100kN, Shimadzu) was utilized for testing elasticity modulus for characterizing mechanical performances at the 1 mm/min loading rate till reaching 80% sample compression. Elasticity modulus was analyzed through determining original linear proportion of stress-strain curve slope.

#### Water contact angle

The contact angle goniometer (LSA100, LAUDA Scientific) was adopted for determining hydrophobicity. The CCD video camera along with lens connected onto the viewing stage was utilized for measuring angle from liquid droplet to solid surface.

#### Morphological analysis

The atomic force microscope (AFM) (SPM9700, Shimadzu) was employed for evaluating PCL and ZIF-8/PCL samples for their microscopic characteristics. The laser scanning confocal microscope (LSCM) (OLS5000, Olympus) was also applied in evaluating melt electrowritten PCL and ZIF-8/PCL samples with regard to the microscopic characteristics.

### Cellular responses to mPCL and mZIF-8/PCL in vitro

#### Biocompatibility test

Cell Counting Kit-8 (CCK-8) assay was employed to examine mineralized PCL (mPCL) and mineralized ZIF-8/PCL (mZIF-8/PCL) scaffolds with regard to their cell growth and cytotoxic activity. Scaffolds were radiated with ultraviolet and sterilized with ozone, followed by immersion within culture medium as well as overnight incubation. Subsequently, this work cultivated rBMSCs using the aforementioned leaching liquor. In proliferation test, this study inoculated 3000 rBMSCs onto the 96-well plates, later 1.6 × 10^5^ rBMSCs were harvested to analyze the cytotoxic activity. Every well was introduced with freshly prepared medium (1 ml) that contained 10% CCK-8 solution (Dojindo) at diverse time (1/2/3/7 days), followed by 2-h incubation. For quantifying cell proliferation, solution absorbance (AD) value was determined at 450 nm with the microplate reader (Thermo).

Calcein AM (live)/PI (dead) was utilized for live/dead staining for determining cell viability. Calcein AM (green) was adopted for live cell staining, while ethidium homodimer-1 (red) for dead cell staining. This work then inoculated cells (2 × 10^5^/well) onto porous scaffold surface (n = 6). Following 2-h original attachment, each scaffold was placed in the novel 24-well plate, followed by addition of 1 mL freshly prepared medium in every well. Plates that contained scaffolds were later subjected to 3/7-day incubation under 37 ℃ with 5% CO_2_ prior to imaging with LSCM. LSCM (A1R, Nikon) was later used to monitor cell adhesion onto scaffold surface. Following 3-h, 12-h, 3-d, and 7-d incubation, PBS was added to wash scaffolds thrice, followed by 30-min fixation with 4% PFA under RT. Cell permeabilization was conducted with 0.5% Triton X-100 for a 5-min period, followed by PBS washing twice, Phalloidin staining for F-actin and DAPI nuclear staining. Finally, the image analyzer (ImageJ, NIH, Bethesda, ML) was employed for quantifying cell perimeter, area as well as Feret’s diameter.

#### Osteogenic differentiation analysis

At 24 h later, the osteogenic induction medium that contained 0.2 mM ascorbic acid, 10^− 7^ M dexamethasone and 10 mM β-glycerol phosphates was added to replace original medium Osteoinductive medium was exchanged at 2-day intervals. At 5- and 14-day after cell culture, i*n-vitro* osteogenesis was examined. qRT-PCR and WB were carried out to analyze osteogenic gene levels within rBMSCs together with osteogenic protein levels onto scaffolds. To analyze extracellular matrix (ECM) mineralization, this work conducted VonKossa staining. After leaching liquor culture, cells and scaffolds were rinsed and immersed within 4% PFA under RT for a 15-min period, followed by 20-min incubation with 1% silver nitrate solution in the presence of UV light.

### Animal responses to mPCL and mZIF-8/PCL scaffolds in vivo

#### Rat critical calvarial defect models and scaffold implantations

For evaluating whether mZIF-8/PCL implants enhanced osteogenesis in vivo, this work utilized 24 6-week-old male adult SD rats weighing 190–230 g (animal center, HUST) and randomized them into three groups, including (i) Blank; (ii) mPCL; and (iii) mZIF-8/PCL (n = 8 rats per group). Before implantation, SBF was added to immerse MEW printed scaffolds for a 7-d period, followed by ethylene oxide–sterilization. Preoperatively, isoflurane and subcutaneous injection of buprenorphine were used for rat anesthesia before making a 3-cm sagittal incision in the scalp center. The 5.0-mm diameter trephine (Nouvag AG, Goldach) was used to drill two parallel critical cranial defects (5.0-mm) in every rat, followed by incision closure following implanting scaffolds in the defect areas.

#### Micro-CT analysis

At 4- and 12-weeks after surgery, rats were killed to dissect the skulls, which were immersed within 10% formalin (n = 8 each). Micro-CT scanning was then conducted to assess bone tissues within defect areas (5-mm and 2-mm in diameter and height, separately). VG studio software was used to reconstruct sample 3D images (average threshold = 226), CTAn was adopted for quantitative morphometric analysis for determining bone volume (BV), bone mineral density (BMD) along with bone volume/tissue volume (BV/TV).

#### Histological and immunohistochemical (IHC) analyses

Sample decalcification within EDTA was carried out for a 4-week period, followed by paraffin embedding, dehydration with gradient ethanol and slicing in 3-µm sections for hematoxylin and eosin (H&E, Beyotime, China) and Masson’s trichrome staining. The expression of OCN was evaluated by IHC analysis with specific antibodies (ab13420, Abcam). Using microscopy, histological and immunohistochemical images were recorded.

### Statistical analysis

Using the Student’s t-test, comparisons of means between groups was carried out whereas one-way analysis of variance (ANOVA) together with the Students’ Newman-Keuls (SNK) post hoc test were adopted for comparing the means between groups. In addition, p ≤ 0.05 was determined to be the threshold for statistical significance.

### Electronic supplementary material

Below is the link to the electronic supplementary material.

Supplementary Material 1
