# Supplementary material for: ZIF-8 induced hydroxyapatite-like crystals enabled superior osteogenic ability of MEW printing PCL scaffolds
Source: J Nanobiotechnology. 2023 Aug 10;21:264. doi: 10.1186/s12951-023-02007-w (PMC10413775; doi:10.1186/s12951-023-02007-w)
Supplement: Supplementary file 1 — Supplementary Material 1 [file 12951_2023_2007_MOESM1_ESM.docx]

Supporting Information

ZIF-8 induced hydroxyapatite-like crystals enabled superior osteogenic ability of MEW printing PCL scaffolds

Bingqian Wang, Yuyang Zeng, Shaokai Liu, Muran Zhou, Huimin Fang, Zhenxing Wang*, Jiaming Sun*

1. **Materials and methods**

**1.1 Preparation of ZIF-8/PLA and evaluation of their mineral properties.**

Electrospun nanofiber membranes were fabricated using a blend of PLA (mean molecular weight 1.6×10^5^, NatureWorks, USA) and ZIF-8 with a ratio of 10:1 in weight. Both PLA and ZIF-8 were dissolved in hexafluoro isopropanol (HFIP) at a total concentration of 8% (wt./vol) and sonication on an ultrasonic water bath for 10min. The solution was delivered by a programmable syringe pump to an electrically charged needle at a flow rate of 1.0 ml/h. The electro-spinning process was performed at a high voltage of 20 kv. Fibers were ejected toward a grounded tinfoil at a distance of 15 cm. The resulting nanofibrous membranes were stored overnight in a vacuum oven to remove residual solvent. The same mineralization experiment conditions were used as described above. Scanning electron microscopy (SEM) images and energy dispersive spectrometer analysis (EDS-Element mapping) were obtained on Gemini 300 (Zeiss, Germany). Samples were sprayed with gold for 15 s and scanned at 5 kV.

**1.2 Preparation of UIO-66/PCL MIL-100/PCL and evaluation of their mineral properties.**

The molten mixture of PCL and UIO-66/MIL-100 (10% wt.) was blended on a 120℃-heating plate for 30 min. The UIO-66/PCL and MIL-100/PCL compacts (10 mm in diameter and 2 mm high) were obtained after pressing.  The same mineralization experiment conditions and SEM testing conditions were used as described above.

1. **Results**


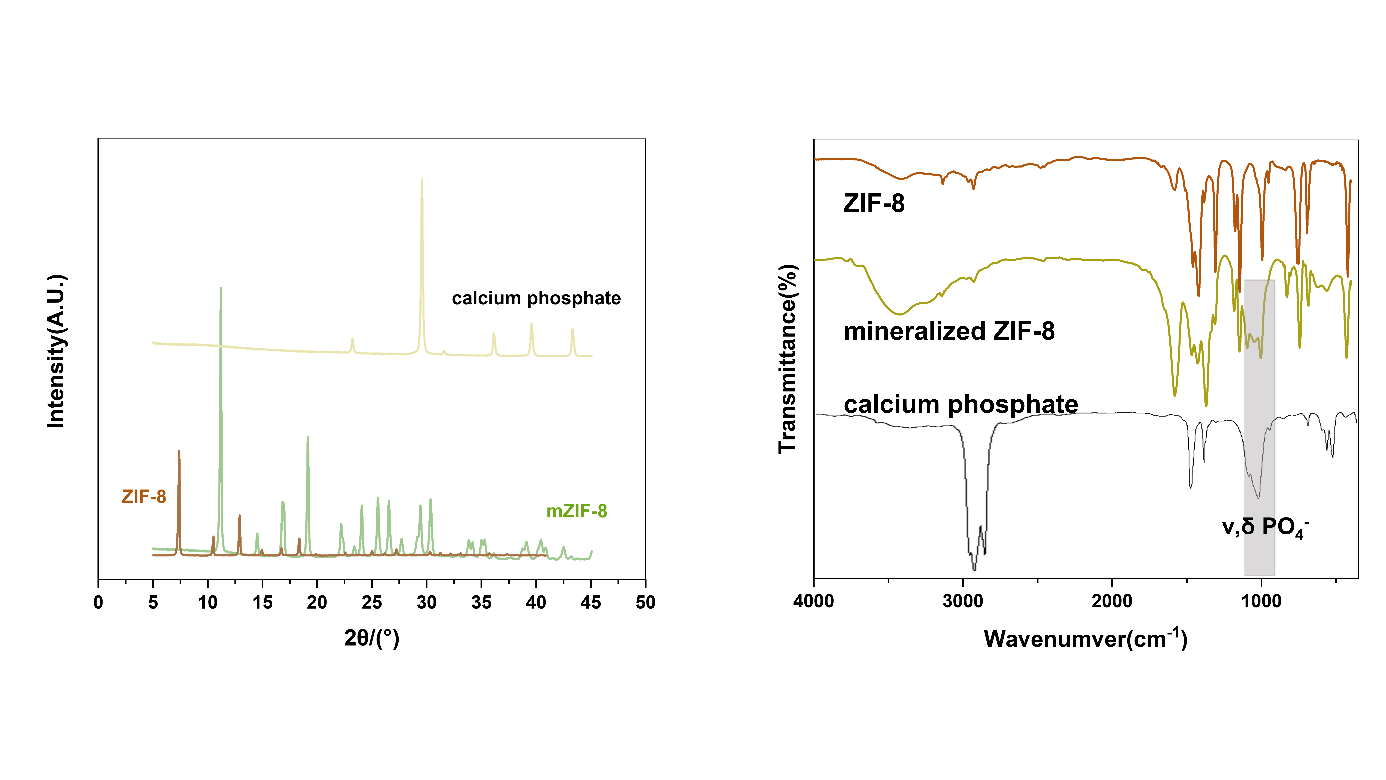


**Figure S1. the XRD and FT-IR spectrum data of nano-hydroxyapatite, ZIF-8 and mineralized ZIF-8.**


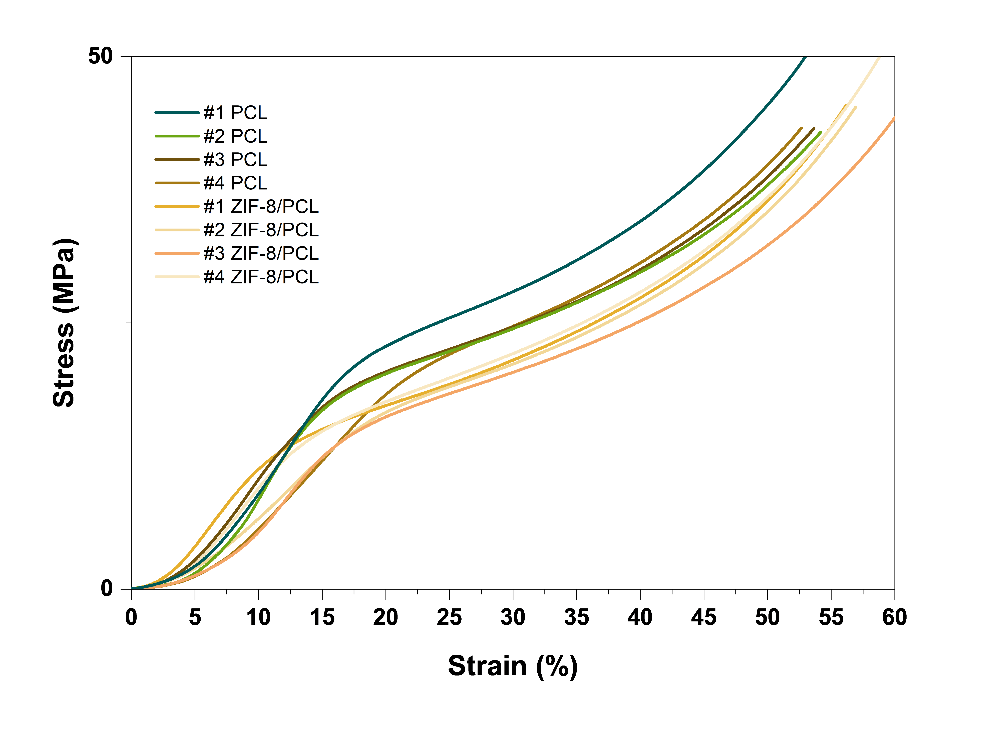


**Figure S2. The initial compressive stress-strain curves of PCL and ZIF-8/PCL.** The slope of the initial linear portion of the stress-strain curves were calculated to obtain the elastic modulus.


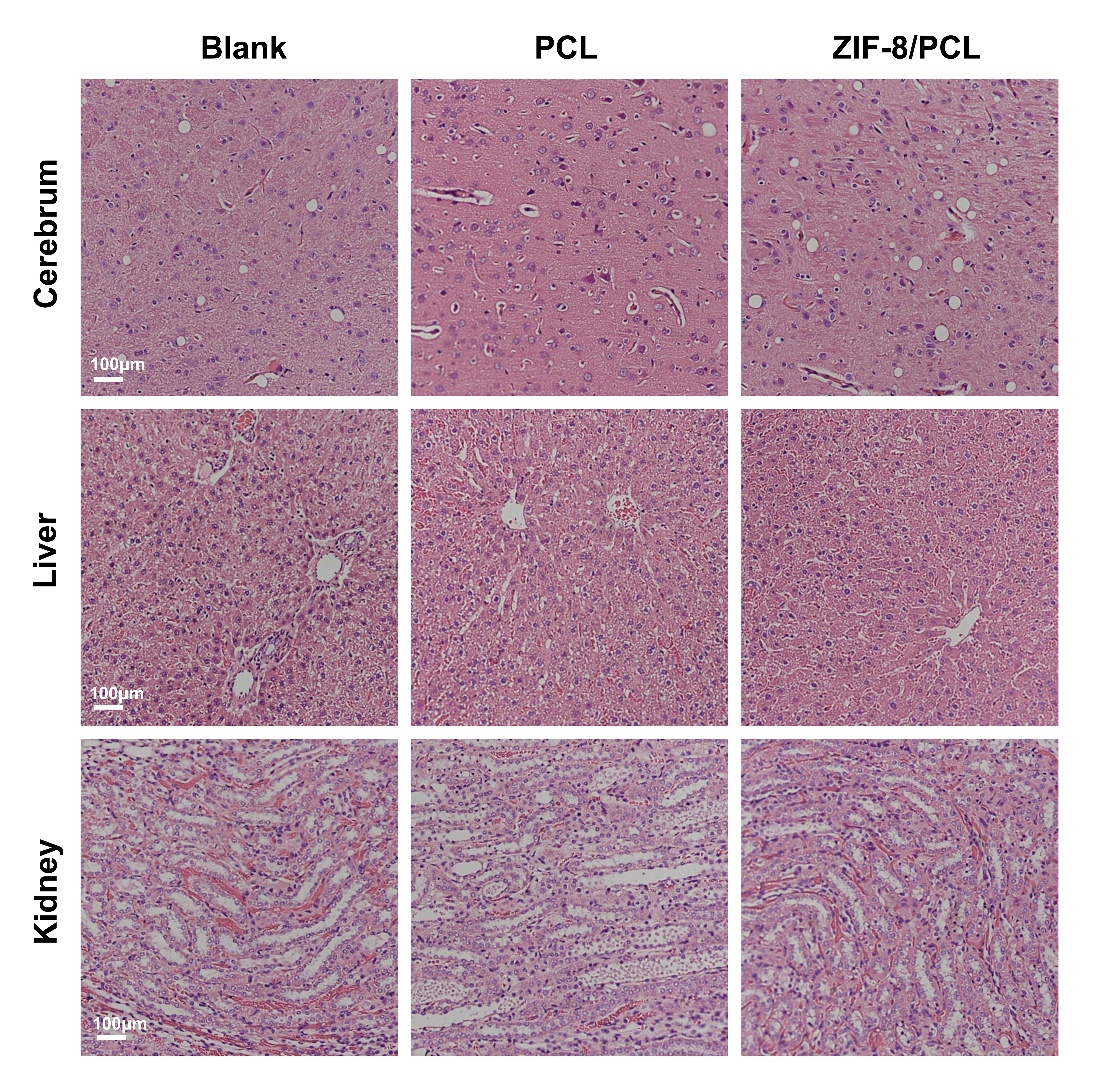


**Figure S3.**  **Histological analysis on major organs.** Cerebrum, liver and kidney sections of the ZIF-8/PCL treated rats appeared histologically normal with no significant pathological changes.


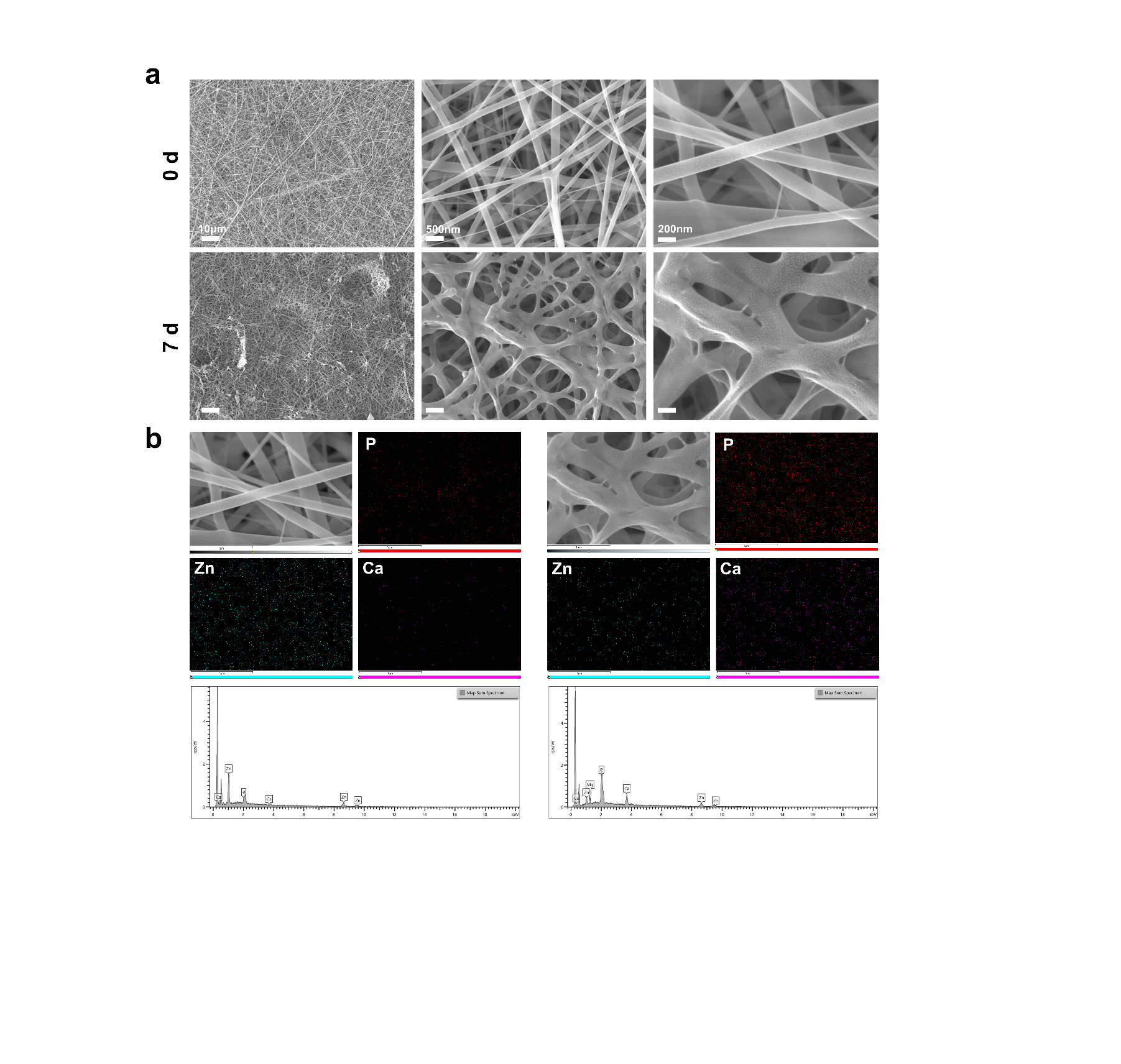


**Figure S4.**  **Characterization of mineralization properties of ZIF-8/PLA.** ZIF-8 was synthesized with PLA by electrospinning. a) SEM images showing mineral deposition on the surface of ZIF-8/PLA electrospun fibers after the 7 days incubation process in SBF, and the diameter of the fibers were increased. b) The elements mapping indicates that the deposition contains Ca, P and Zn elements.


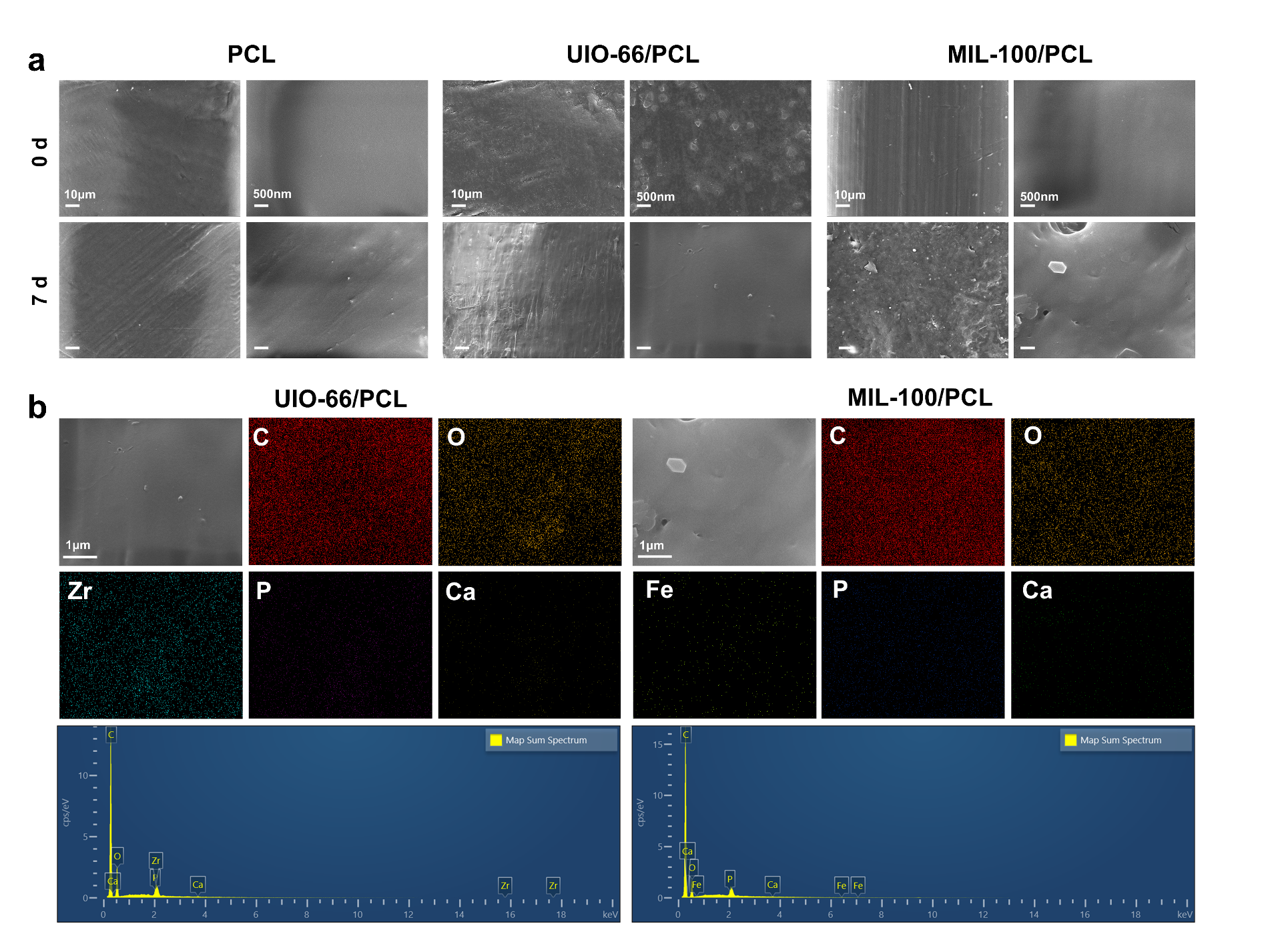


**Figure S5.**  **Characterization of mineralization properties of PCL combined with other subtypes MOFs.** The mineralization properties were all studied in simulated body fluid (SBF) over a total period of 7 days. a) No noticeable surface topography changes were observed in SEM images, suggesting that MIL-100, UIO-66 do not exhibit mineralization ability. b) The elements mapping indicates that the there was no mineral deposition.
